# Phase I clinical trial of AXL1717 for treatment of relapsed malignant astrocytomas: analysis of dose and response

**DOI:** 10.18632/oncotarget.20662

**Published:** 2017-09-06

**Authors:** Robert Aiken, Magnus Axelson, Johan Harmenberg, Maria Klockare, Olle Larsson, Cecilia Wassberg

**Affiliations:** ^1^ Rutgers-Cancer Institute of New Jersey, New Brunswick, NJ, U.S.A; ^2^ Clinical Chemistry, Department of Molecular Medicine and Surgery, Karolinska University Hospital and Karolinska Institutet, Stockholm, Sweden; ^3^ Axelar AB, Karolinska Institutet Science Park, Solna, Sweden; ^4^ Cellular and Molecular Tumor Pathology, Department of Oncology and Pathology, Cancer Centre Karolinska, Karolinska University Hospital and Karolinska Institutet, Stockholm, Sweden; ^5^ Section of Radiology and Nuclear Medicine, Department of Molecular Medicine and Surgery, Karolinska University Hospital and Karolinska Institutet, Stockholm, Sweden

**Keywords:** IGF-1R, phase 1 clinical trial, mitotic catastrophe, signal transduction, tyrosine kinase inhibitor

## Abstract

**Purpose:**

Early phase I study of safety of AXL1717 in patients with recurrent or progressive malignant astrocytomas and evaluation of preliminary anti-tumor efficacy.

**Patients and methods:**

Nine patients fulfilling the set criteria were enrolled. Eight had recurrent glioblastoma and one gliosarcoma. Patients were treated with an oral suspension of AXL1717 (215-400 mg bid) cycle-by-cycle in 35-day cycles (28 days bid and 7 days off). Patients with progressive disease and/or toxicity-related dose delay of more than 14 days were withdrawn.

**Results:**

Four patients had tumor responses (44%) to AXL1717 treatment. Two of these had stable disease for 12 months (10 cycles at 215-300 mg bid). Due to MRI-detected progression they were then taken off the study. They died 8 and 12 months later, respectively. One patient was treated 8 months (6 cycles with 215 mg bid). He was withdrawn because of disease progression but died after another 25 months. The fourth patient having stable disease died of sepsis due to pancytopenia in the end of cycle 2 on 400 mg bid. A fifth patient underwent surgery after two cycles with 300 mg bid. Pathological analysis demonstrated abundant necrosis and small areas of viable tumor. After one more cycle with 300 mg bid he was withdrawn due to clinical and radiographic worsening and died 11 months later. The other 4 patients did not have any detectable responses and died within 3-13 months after trial entry. Neutropenia was the main adverse effect, which was easily detected and reversible in all but one patient.

**Conclusion:**

This clinical phase I study indicates that AXL1717 as a single agent is capable of producing prolonged stable disease and survival of patients with relapsed malignant astrocytomas. The drug was well tolerated. A new formulation of the drug will be used in further investigations in order to better define the optimal dose.

## INTRODUCTION

Despite revolutionary advances in brain imaging, operative neurosurgery, and conformal radiation dosing and delivery, there has not been a commensurate improvement in survival of patients who have glioblastomas and other malignant astrocytomas, the median survival after diagnosis being about 15 months. These tumors are pathologically characterized by high mitotic rate, hypercellularity, necrosis, and vascular endothelial proliferation. Numerous signal transduction pathways have been implicated in the pathogenesis of these tumors and/or their transformation into tumors which possess more malignant and invasive properties. The insulin-like growth factor-Type I receptor (IGF-1R) and its cognate ligands IGF-1, IGF-2, and insulin, have been strongly implicated as an important pathway in the evolution of astrocytic neoplasms [1]. Recently, it was confirmed that IGF-1R is overexpressed in glioblastomas and has been identified as an independent prognostic factor that is characterized by shorter survival and was associated with a less favorable response to temozolomide [2].

In 1996, Resnicoff and co-workers [3] demonstrated that C6 glioma cells expressing an antisense IGF-1R RNA were no longer tumorigenic in syngeneic rats, protected them from subsequent tumor re-challenge, and caused regression of established subcutaneous tumors. The efficacy of this strategy was further investigated in intracerebrally implanted C6 rat glioblastoma cells. They demonstrated that C6 tumor cells expressing an antisense IGF-1R RNA implanted for 24 hours in the subcutaneous tissue of rats evoked an anti-tumor response in the brain leading to complete brain tumor regression and long-term survival of the rats (abscopal effect) [3]. Employing this strategy, they developed an antisense ODN (oligodeoxynucleotide) against the IGF-1R and in a Phase 0 human clinical trial demonstrated that *ex-vivo* IGF-1R antisense ODN treatment with patient derived autologous glioma cells safely induced apoptosis and a host response [4]. Of 12 patients who participated in the trial, 8 had clinical and radiographic responses and 3 patients had complete radiographic disappearance of their tumors (1 recurrent anaplastic astrocytoma, 2 recurrent glioblastomas). The project was eventually suspended because additional GMP lots of the antisense ODN targeting the IGR-1R could not be manufactured reliably.

The semisynthetic cyclolignan picropodophyllin (PPP), the active agent in AXL1717, is an orally available small molecule of 414 MW, that inhibits IGF-1R signaling without any apparent effect on the highly homologous insulin receptor (InR) [5, 6]. In 2010, Yin et al. demonstrated that PPP inhibited growth of human glioblastoma cell lines, reducing phosphorylation of IGF-1R and AKT [7]. *In vivo* PPP caused dramatic tumor regression not only in subcutaneous human xenografts on SCID mice but also in intracerebral human xenografts on nude rats which implicated passage of PPP across the blood-brain-barrier [7].

More recently, Osuka et al. [8] showed that fractionated radiation of mouse glioma stem cells induced radioresistance through increased secretion of IGF-1 and upregulation of IGF-1R. Tumors formed from these cells were also confirmed to be radio-resistant *in vivo*, but they were substantially growth inhibited by monotherapy with PPP. Interestingly, PPP treatment also made the tumors radiosensitive [8].

A recent clinical phase I study on patients with various advanced cancers demonstrated that AXL1717 (PPP in an oral suspension for human use) was well tolerated with neutropenia as the only dose-related adverse effect and that the neutropenia was reversible [9]. Furthermore, the drug exhibited a promising anti-tumor effect in this heavily pretreated patient cohort.

The main objective of this early phase 1 clinical trial was to ascertain the safety and tolerability of AXL1717 in patients with recurrent or progressive malignant astrocytomas who previously failed at least one standard therapy. A secondary objective was to evaluate preliminary evidence of anti-tumor efficacy.

## RESULTS

### Patient characteristics and efficacy

The clinical trial enrolled nine patients from December 2012 to October 2013. A 10^th^ patient was excluded from the study after screening but before treatment and is not considered in this analysis. At the time of entry into the clinical trial, eight patients had glioblastoma and one had gliosarcoma, a glioblastoma subtype consisting of both gliomatous and sarcomatous components [10, 11] (Table 1). The status of the prognostic glioma biomarkers O (6)-methylguanine-DNA methyltransferase (MGMT) promoter (methylated or unmethylated), isocitrate dehydrogenase 1 and 2 (IDH1/2) (wild-type or mutated), phosphatase and tensin homolog (PTEN) (wild-type or deleted), and epidermal growth factor receptor (EGFR) (amplified or mutated) in the tumors are presented in Table 1. Drug compliance exceeded 95% evidenced by the completed diaries of the patients. Best responses consisted of stable disease (clinical and imaging) seen in four patients (Patients 3, 5, 6, and 8 treated for 2, 10, 10, and 6 cycles, respectively) as shown in Table 2. In one other patient (Patient 4), the unusual tumor response described below was also seen. The survival time of all the patients is also shown in Table 2.

**Table 1:** Patient characteristics

Patient	Age and Gender	Tumor type^1^	# FailedTherapies before AXL1717	MGMT promoter(methylationStatus)	IDH 1/2(wild-type/mutated)	PTEN(wild-type/deleted)	EGFR(amplified/mutated)
1	57 F	GBM	1	+	Wild-type	Deleted	vIII mutated
2	59 M	GBM	2	+	Wild-type	Deleted	Not amplified
3	58 F	GBM	2	NT^2^	NT^2^	Deleted	Amplified
4	57 M	AA → GBM	2	-	Wild-type	Deleted	NT^2^
5	69 M	GBM	1	+	Wild-type	Deleted	Not amplified
6	37 M	GBM	1	+	Wild-type	Deleted	Not amplified
7	61 F	GBM	1	-	Wild-type	Wild-type	Not amplified
8	53 M	GSC	3	+	Wild-type	Wild-type	Not amplified
9	59 M	GBM	3	+	Wild-type	Wild-type	Not amplified

^1^GBM = Glioblastoma; AA = Anaplastic astrocytoma; GSC = Gliosarcoma.

^2^NT = Not tested.

**Table 2:** AXL1717-Treatment, response and survival of patients

Patient	Dose bid(mg)	# Cycles received	Response^1^	Survival time^2^(months)
1	400	1^3^	PD	13
2	400	2^3^	PD	3
3	400^4^	2^3^	SD	2
4	300	3	PR	15
5	215^4^	10	SD	20
6	300	10	SD	24
7	300	1	PD	6
8	215^4^	6	SD	33
9	300	1	PD	3

^1^PD = Progressive disease; SD = Stable disease; PR = Partial regression.

^2^Months from start of AXL1717-treatment.

^3^Includes one incomplete treatment cycle. A total cycle = 1.2 months.

^4^Starting dose 300 mg bid.

Patient 3, a 58-year old woman with a recurrent GBM (failed temozolomide (TMZ) + intensity-modulated radiation therapy (IMRT), monthly TMZ, and then bevacizumab after TMZ failure) showed stable disease on imaging and clinical function during the first cycle with AXL1717, 300 mg bid, but developed febrile neutropenia and died of gram negative sepsis at the end of cycle 2, on the dose 400 mg bid.

**Figure 1:**
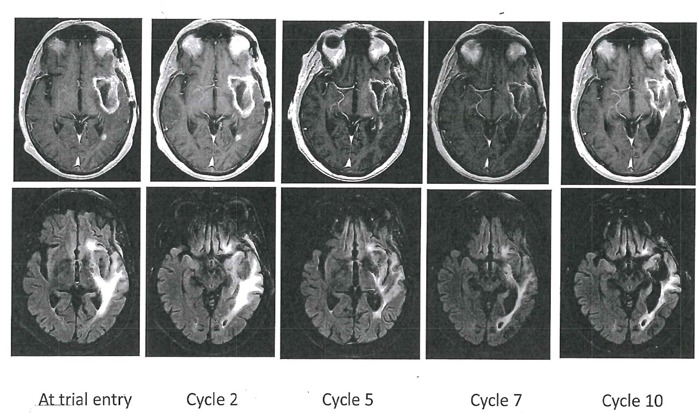
Axial T1-weighted contrast-enhanced (top row) and T2-FLAIR images (bottom row) in Patient 5 This patient had a left temporal glioblastoma illustrated as a cystic lesion with a rim of contrast-enhancement. This cavity was stable throughout the serial MR examinations and T2-FLAIR showed decreased edema. Four smaller contrast-enhancing lesions (out-of-field) were also stable throughout the study in the left temporal and left periventricular region. After cycle 7, a small new contrast-enhancing lesion appeared in the left temporal region and represented progressive disease.

Patient 5, a 69 year-old man with a recurrent GBM (IDH1/2 wild type, methylated MGMT promoter), had tumor progression on a clinical trial consisting of IMRT/TMZ, then TMZ and vorinostat (a histone deacetylase inhibitor). He developed grade 3 neutropenia on AXL1717, 300 mg bid after 2 cycles. Thereafter, the dose was reduced to 215 mg bid which he tolerated well and had stable clinical and imaging disease of left temporal and periventricular lesions (see serial MRIs, patient 5 in Figure 1). Additionally, T2 FLAIR sequences showed decreased edema during the serial MRI examination. Evaluation of his MRI imaging demonstrated re-enhancement of a pre-existing portion of the tumor in the left periventricular region suggesting tumor progression after cycle 7. He was taken off study after 12 months treatment (cycle 10) although clinically stable. He died 8 months later of disease progression.

**Figure 2:**
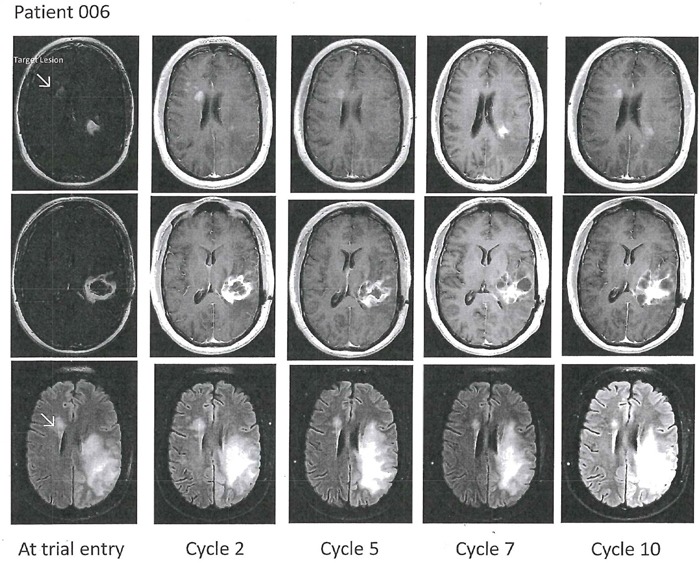
Axial T1-weighted contrast enhanced (top two rows) and T2-FLAIR images (bottom row) in Patient 6 with an original left temporal glioblastoma as depicted in the middle row The patient developed a right frontal periventricular tumor progression, marked with the arrow as the index tumor at baseline. Follow-up images showed stable disease of the index tumor and as for the left temporal tumor component evolving multi-cystic contrast-enhancing changes and edema were noted.

Patient 6, a 37 year-old man, initially had partial resection of a left temporal glioblastoma (IDH1/2 wild type, methylated MGMT promoter). He developed new right frontal tumor involvement on a Phase 1/2 clinical trial of IMRT/TMZ + trans-sodium crocetenate (TSC), a putative radiation sensitizer. He was qualified to the AXL1717 trial because of the un-irradiated new right frontal index tumor remote from his radiated tumor and received 300 mg AXL1717 bid for 10 cycles, which he tolerated without any hematological adverse effects. The serial MRI examinations of patient 6 are shown in Figure 2. Evaluation of imaging disclosed a new small tumor lesion (< 5 mm) and subsequent stable contrast-enhancing lesion consistent with recurrence in the original left temporal tumor after cycle 8. He was taken off the trial after 12 months (cycle 10) because of this observation and tumor progression. He remained clinically high performing (KPS 100) throughout the entire trial period. He died 12 months later of disease progression.

Patient 8 (imaging not displayed), a 53 year-old man, originally had a right frontal gliosarcoma (IDH1/2 wild type, methylated MGMT promoter). He failed prior treatment including IMRT/TMZ, and monthly TMZ on a clinical trial (Immunocellular, ICT-107). At the time of entry into this clinical trial, he had several progressing frontal and temporal contrast-enhancing tumors on MRI. Treatment with AXL1717 began with 300 mg bid but he developed grade 3 neutropenia after the first cycle and the dose was reduced to 215 mg bid. He had intermittent grade 2 neutropenia also at 215 mg bid necessitating occasional G-CSF and was taken off trial after 8 months treatment (cycle 6) because of the recurrent neutropenia. He was clinically and imaging stable (KPS 100) throughout the trial period. He died 25 months later of disease progression.

**Figure 3:**
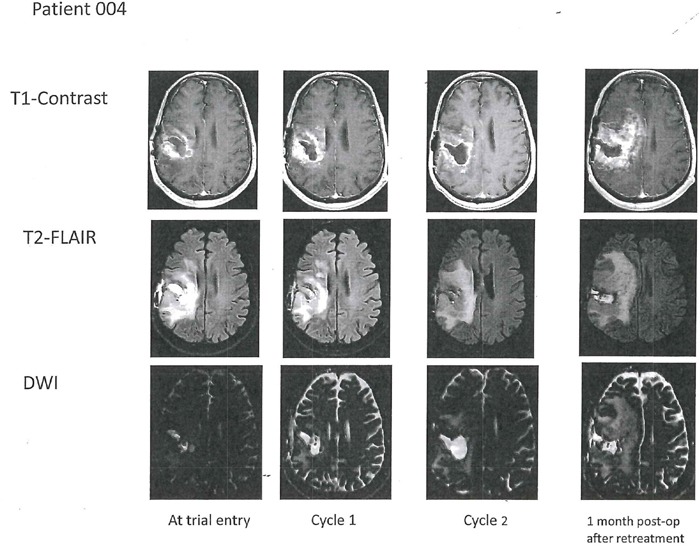
Axial T1-weighted contrast-enhanced images (top row) in Patient 4 with right temporal glioblastoma at trial entry with corresponding T2-FLAIR (middle row) and corresponding DWI with apparent diffusion coefficient (ADC) sequences (bottom row) After two cycles with AXL1717 there was enlargement of the tumor cavity. Surgical resection histopathology showed abundant necrosis with small islands of viable tumor. Progressive disease on imaging (and clinically) after an additional treatment cycle (post-operative; last column) and the patient was removed from the trial. T2 FLAIR shows increased edema post-operatively one month after treatment.

Patient 4 was a 57 year-old man whose original tumor was an IDH1/2 wild type anaplastic astrocytoma with unmethylated MGMT promoter and PTEN deletion (Table 1). At trial entry, re-operation demonstrated that the tumor had become a glioblastoma. There were some residual areas of treatment effect. After cycle 1 on AXL1717, 300 mg bid, there was evolution of “salt and pepper” peri-tumoral contrast-enhancement on MRI that was worse after the second cycle necessitating an increase in his dexamethasone dose. (See Patient 4 serial MRI examinations in Figure 3). Re-operation/tumor re-resection histopathology demonstrated abundant areas of tumor necrosis and small areas of viable tumor. Because of this encouraging result he was treated with a third cycle of AXL1717, 300 mg bid, but was then taken off the study when he developed further clinical decline. He died 11 months later with MRI suggestive of progressive disease. An autopsy was not performed.

Patients 1, 2, 7, and 9 did not show any clinical or imaging responses (Table 2).

### Safety and tolerability

The main serious adverse events (SAE) consisted of neutropenia and were seen in five patients. In two of the first treated patients thrombocytopenia also developed (Tables 3 and 4). The first three patients were treated with the dose 400 mg bid. One of these patients (Patient 3), a 58 year-old woman with a right parietal glioblastoma developed febrile neutropenia and died of gram negative sepsis in week 3 of the second cycle due to the investigational agent. After this incident the dose of AXL1717 given to patients was reduced from 400 mg bid to 300 mg bid or lower and no episodes of sepsis occurred subsequently. Neutropenia often occurred in cycle 1 or 2 and, although reversible, prompted a protocol mandated dose reduction. One patient (Patient 8) had episodes of neutropenia (grade 2) also at the lowest dose used in the trial, i.e. 215 mg bid. Paresthesias (grade 1) in Patient 6 was the only non-hematological AE observed (Table 3). Hyperglycemia, nausea, vomiting, diarrhea and constipation were not observed in any of the patients. Similarly, there was no effect on renal, hepatic, or cardiac function. Within the limited context of this clinical trial AXL1717 was well tolerated.

**Table 3:** Adverse events (AE/SAE) by grade

AE/SAE	Grade		
	1-2	3-4	5
Anemia		1	
Neutropenia	1	5	
Thrombocytopenia		2	
Sepsis			1
Paresthesias	1		

**Table 4:** Adverse events (AE/SAE) by patient

Patient	AE/SAE and grade
1	Grade 4 anemia, neutropenia and thrombocytopenia. Did not recover ≤ Grade 2 in 2 weeks, off study
2	None
3	Grade 4 neutropenia and thrombocytopenia at 400 mg bid; Grade 5 sepsis
4	Grade 3 neutropenia at 300 mg bid
5	Grade 3 neutropenia at 300 mg bid; none at 215 mg bid
6	Grade 1 paresthesias
7	None
8	Grade 3 neutropenia at 300 mg bid; Grade 2 at 215 mg bid, off study
9	None

## DISCUSSION

Malignant astrocytomas remain lethal cancers despite considerable advances in neurosurgery, highly conformal radiation techniques, and chemotherapy. Median survival after diagnosis remains a stagnant 15 months. Temozolomide is used for first-line treatment, but almost all patients with glioblastomas, the most common of malignant primary brain tumors, relapse due to development of drug resistance. When relapse occurs, treatment options are very limited and the response is generally short. Bevacizumab prolongs overall survival only by 4-6 months. In a recent phase 3 study (CheckMate -143, see https://immuno-oncologynews.com/2017/04/18/opdivo-fails-demonstrate-increased-survival-glioblastoma-multiforme-phase-3-study/) patients with recurrent glioblastoma were treated with the immune-checkpoint inhibitor Opdivo (nivolumab). However, also with this treatment there was no prolongation of survival of the patients compared to standard-of-care treatment (bevacizumab). So far no pharmaceutical agent or regimen has shown any phase 3-supported survival benefits over bevacizumab in patients with recurrent glioblastoma.

This is the first clinical trial of AXL1717 in patients with relapsed malignant astrocytomas. We are reporting here our experience using AXL1717 as an oral suspension. A new oral formulation (capsule) offering a higher dose-exposure linearity of the drug is under development and will be used in future clinical trials in order to define an optimal dose regimen for AXL1717, including its recommended phase 2 dose.

Four out of nine patients in the present study had stable disease when treated with AXL1717. Three patients had prolonged stable disease and overall survival of 20-33 months when including the trial period, which corresponds to 8-25 months after discontinuation of AXL1717 treatment. Even when radiographic progression was detected, minimal disease progression occurred in subsequent MRI examinations and patients remained clinically stable. In addition, one other patient (Patient 4) developed a “salt and pepper” MRI appearance of his tumor after treatment with AXL1717, indicating areas of bleeding and necrosis typical for highly vascular tumors. The tumor was found to have large areas of tumor necrosis and small residual areas of viable tumor at the time of surgical re-resection after 2 treatment cycles that suggested pseudoprogression.

Neutropenia and thrombocytopenia, grade 3-4, were the major dose limiting side effects in this cohort and, when present, often developed early in treatment. This may be a class effect of IGF-1R targeted inhibitors [12]. Thrombocytopenia was seen in two of the first treated patients, receiving the highest dose of AXL1717, 400 mg bid, whereas neutropenia could also be seen in some patients treated with lower doses. In fact, one patient developed episodic neutropenia when treated with the lowest dose used in the trial, 215 mg bid. Interestingly, this patient survived the longest of all patients, in total 33 months from entering the trial, corresponding to 25 months after trial. This may indicate that the patient was super sensitive or extra-exposed to the drug when treated. Grade 1 paresthesias developed in patient 6, who received 10 cycles of treatment without neutropenia and thrombocytopenia. This non-hematological effect occurring during the first cycle of AXL1717 has not been previously observed [9]. No patient developed hyperglycemia, suggesting that the insulin receptor is not a clinically significant target of AXL1717 in contradistinction to the IGF-1R inhibitor OSI-906 and the IGF-1R targeting monoclonal antibodies [13]. Overall AXL1717 as an oral suspension was well tolerated. Anorexia, nausea, vomiting, diarrhea, constipation and dermatologic derangements attributable to the investigational drug did not occur in any of the study patients. Furthermore, no effect on renal, hepatic or cardiac function was seen. When neutropenia developed, it was generally reversible and easy to monitor.

The exact mechanism(s) of action of AXL1717 (i.e. PPP) are still unknown. Many studies in various cell lines and animal tumor models have demonstrated that PPP inhibits IGF-1R activity or its downstream signaling pathways. In some studies not only inhibition of IGF-R phosphorylation was observed, but also downregulation of the receptor [14]. Consistently, in the phase I trial performed by Ekman et al. [9] the cell surface IGF-1R expression in granulocytes of patients was reduced by up to 40% upon treatment with AXL1717. Furthermore, serum levels of IGF-1 and IGFBP3 increased significantly after 14 days of treatment. Together, these findings are compatible with IGF-1R targeting. In contrast, levels of insulin and C-peptide in serum, and glucose and HbA1c in blood, remained unchanged [9], indicating that the InR-mediated metabolic signaling is not attenuated by AXL1717 in humans. In a related matter, Rostoker et al. [15] compared the effects of the InR inhibitor S961 and PPP on tumor growth and metabolism in a hyperinsulinemic mouse model. Whereas S961 treatment increased tumor growth as related to vehicle control, PPP substantially inhibited it. The blood glucose and serum insulin levels were strongly increased by the InR inhibitor, but were unaffected and only slightly increased, respectively, by PPP [15]. Thus, PPP seems to have a minute metabolic effect in these mice.

In addition to the inhibitory effect of PPP on IGF-1R signaling, the compound has been found to cause cell cycle arrest in mitosis in a way resembling mitotic catastrophe [16]. The arrest was observed in neoplastic cell lines, including hepatocellular cancer cells and tumor xenografts, but not in normal hepatocytes and mouse tissues. The effect is not IGF-1R dependent but affects tubulin by an unknown mechanism [16]. Microtubule inhibition represents a mechanism of action of commonly used anti-cancer pharmaceuticals. A major limitation of them is the high rate of neurotoxicity [17]. Notably, none of the 49 patients in the phase I trial with AXL1717 showed any neurological side effects, despite that very high doses (up to 2900 mg bid in dose finding studies) were given [9]. Thus, the mild paresthesias (grade I) seen in one patient in the current study may not directly be linked to AXL1717. This suggests that the mitotic arresting effect of PPP differs significantly from the effects of previously known microtubule inhibitors.

Although the mitotic arrest effect of AXL1717 may contribute to the hematological AEs observed in this and the previous study by Ekman et al. [9], this mechanism of action of AXL1717 in combination with IGF-1R inhibition may be favorable, or even crucial, for the anti-tumor efficacy of the drug.

In conclusion, this phase I clinical trial reveals that AXL1717 is a promising drug having the capability as a single agent to produce sustained clinical responses in patients with relapsed malignant astrocytomas. Further investigations, using a new oral formulation of AXL1717, will better define the optimal dose and mechanism(s) that are operational.

## MATERIALS AND METHODS

### Study organization

This clinical trial was an investigator-initiated study (RDA was the Principal Investigator, who also held the IND: 114412; http://clinicaltrials.gov NCT01721577). It was conducted at a single-center. The protocol, informed consent, and all amendments were reviewed and approved by the institution's IRB in accordance with the Declaration of Helsinki and International Conference for Harmonization of Good Clinical Practices guidelines. All patients provided voluntary written informed consent.

### Patients

Patients with histologically verified recurrent or progressive anaplastic astrocytoma, anaplastic oligodendroglioma, anaplastic ependymoma, glioblastoma (GBM), or gliosarcoma who had received at least one accepted standard therapy were allowed to enter the clinical trial. Inclusion criteria were age ≥ 18 years of age, life expectancy of at least 3 months, KPS ≥ 60 or performance status ECOG ≤ 2, essentially normal clinical laboratory tests, including hematology parameters, blood and urine chemistry (e.g. liver and renal function tests), coagulation status, as well as a 12-lead EKG with normal tracing or changes that were not clinically significant, including QTc <500 ms. Patients had to be at least 2 weeks from cytoreductive surgery, if performed, 4 weeks from bevacizumab or other chemotherapy, if given (6 weeks if the prior therapy was a nitrosourea), at least 12 weeks from completion of radiation therapy unless the relapse occurred outside of the radiated site, and be at least 7 days off medications which inhibit or induce CYP2C9 or CYP3A4 before first study treatment day. Patients had to be able to undergo serial MRI scans with and without contrast as defined in the protocol.

Exclusion criteria included grade 3 or higher constipation within the past 28 days or grade 2 constipation within the past 14 days. This is because AXL1717 absorption from the gut can be markedly enhanced in the setting of significant constipation [9]. Other exclusion criteria included positive Hepatitis B or C serology, HIV infection requiring anti-retroviral therapy, medically uncontrolled Type 1 or Type 2 diabetes mellitus, prior stereotactic or gamma knife radiosurgery or proton radiation, unless there was unequivocal progression by functional neuro-imaging (PET, diffusion weighted MRI, MR spectroscopy, MRS, SPECT) or re-operation with documented histologic confirmation of recurrence, prior intra-tumoral chemotherapy or systemic immunotherapy unless unequivocal progression documented by functional neuro-imaging or by re-operation with documented histologic confirmation of recurrence. Furthermore, pregnancy, lactation, or participation in any other investigational clinical trial if ≤ 8 weeks from last treatment, and known or suspected hypersensitivity to the investigational agent or other suspension components led to exclusion.

### Study design

The study was an open-label, single-center, sequential, multi-dose, phase 1 study that consisted of a 3 + 3 dose escalation schedule. The treatment regimen in the present trial was adopted from a previous clinical phase I trial with AXL1717 on patients with various cancers [9], since safety, pharmacokinetic and pharmacodynamic data on the patients at different dose levels were reported. The initial dose chosen, 400 mg twice daily given orally, was well tolerated in that trial, but exceeded the MTD of the patients in this trial and was subsequently de-escalated. Since some patients developed grade 3-4 hematologic toxicity also at the dose of 300 mg twice daily, the dose was further reduced to 215 mg twice a day.

The drug AXL1717 was given to patients twice per day as an oral suspension. At each dose occasion, patients were instructed to take the AXL1717 suspension with a meal because absorption increased several-fold with food. The treatment schedule consisted of 28 consecutive days on treatment (the dose taken twice daily on days 1-28) followed by 7 days off treatment (days 29-35) which was the treatment schedule adopted from Ekman et al [9]. The rationale for this treatment regimen was to continuously suppress cellular IGF-1 signaling for a long time leading to tumor cell death, and was based on the initial assumption that AXL1717 only inhibits IGF-1R. The patients in our trial were evaluated weekly by physical examination and adequate laboratory and other tests. MRI brain imaging protocol for 1.5 Tesla included T1-weighted pre- and post-contrast imaging for parenchymal enhancement and perfusion, T2-weighted imaging and T2-FLAIR for edema, diffusion weighted imaging (DWI) and multi-voxel magnetic resonance spectroscopy (MRS). Imaging examinations were performed at the beginning of the off-treatment week of every cycle, usually on day 28-29. Pharmacologic use of corticosteroids was permitted at the lowest necessary dose. Hematopoietic growth factors and/or erythropoietin-stimulating factors could be used, if needed, for acute grade 3-4 toxicity, but not for routine prophylaxis. All women of child bearing potential and sexually active men agreed to use effective contraception. Subjects with a serious co-morbid medical condition, or a clinically significant laboratory finding(s) or any finding(s) on history or examination that, in the opinion of the Investigator, interfered with the conduct of the study or could put the subject at unacceptable risk, were excluded. Clinical and radiographic responses were determined after every cycle (approximately every 5 weeks). Imaging treatment response assessment was performed according to the RANO criteria (Response Assessment in Neuro-Oncology) [18]. Adverse events were graded using CTCAE version 3.0 and classified according to relationship to treatment (definite, probable, possible, unlikely, or unrelated). Hematologic DLT was defined by: grade 4 neutropenia (ANC) of > 7 days, grade 4 thrombocytopenia of > 7 days or grade 4 anemia of > 7 days. Toxicity related dosing delay of > 14 days required that the patient withdraw from the study.
